# Rapid Selection and Proliferation of CD133(+) Cells from Cancer Cell Lines: Chemotherapeutic Implications

**DOI:** 10.1371/journal.pone.0010035

**Published:** 2010-04-08

**Authors:** Sarah E. Kelly, Altomare Di Benedetto, Adelaide Greco, Candace M. Howard, Vincent E. Sollars, Donald A. Primerano, Jagan V. Valluri, Pier Paolo Claudio

**Affiliations:** 1 Department of Biochemistry and Microbiology, Joan C. Edwards School of Medicine, Marshall University, Huntington, West Virginia, United States of America; 2 Department of Basic and Applied Biology, Faculty of Sciences, University of L'Aquila, L'Aquila, Italy; 3 CEINGE-Advanced Biotechnology, s.c.ar.l., Naples, Italy; 4 Department of Biomorphological and Functional Science, University of Naples “Federico II”, and IBB-CNR, Naples, Italy; 5 Department of Biology, Marshall University, Huntington, West Virginia, United States of America; 6 Department of Surgery, Joan C. Edwards School of Medicine, Marshall University, Huntington, West Virginia, United States of America; Harvard Medical School, United States of America

## Abstract

Cancer stem cells (CSCs) are considered a subset of the bulk tumor responsible for initiating and maintaining the disease. Several surface cellular markers have been recently used to identify CSCs. Among those is CD133, which is expressed by hematopoietic progenitor cells as well as embryonic stem cells and various cancers. We have recently isolated and cultured CD133 positive [CD133(+)] cells from various cancer cell lines using a NASA developed Hydrodynamic Focusing Bioreactor (HFB) (Celdyne, Houston, TX). For comparison, another bioreactor, the rotary cell culture system (RCCS) manufactured by Synthecon (Houston, TX) was used. Both the HFB and the RCCS bioreactors simulate aspects of hypogravity. In our study, the HFB increased CD133(+) cell growth from various cell lines compared to the RCCS vessel and to normal gravity control. We observed a (+)15-fold proliferation of the CD133(+) cellular fraction with cancer cells that were cultured for 7-days at optimized conditions. The RCCS vessel instead yielded a (−)4.8-fold decrease in the CD133(+)cellular fraction respect to the HFB after 7-days of culture. Interestingly, we also found that the hypogravity environment of the HFB greatly sensitized the CD133(+) cancer cells, which are normally resistant to chemo treatment, to become susceptible to various chemotherapeutic agents, paving the way to less toxic and more effective chemotherapeutic treatment in patients. To be able to test the efficacy of cytotoxic agents in vitro prior to their use in clinical setting on cancer cells as well as on cancer stem cells may pave the way to more effective chemotherapeutic strategies in patients. This could be an important advancement in the therapeutic options of oncologic patients, allowing for more targeted and personalized chemotherapy regimens as well as for higher response rates.

## Introduction

Neoplasms may be viewed as tissue consisting of a heterogeneous population of cells that differ in biological characteristics and potential for self-renewal [1]. The clonal nature of certain malignant tumors is well established [2]. According to the model of clonal evolution of tumor cells, cancer is formed through the accumulation of genetic changes in cells and gradual selection of clones [3], [4]. Therefore, the tumor is regarded as abnormal tissue that descended from a single cell through continuous accumulation of genetic errors and various epigenetic changes. However, several experiments carried out during the last decades have shown that not every tumor cell is a tumor initiating cell (T-IC) and that as many as 10^6^ murine or human tumor cells are required to transplant a new tumor from an existing one [4], [5], [6], [7]. This evidence suggested the possibility that tumor cells may exist in a hierarchical state in which only a small number of cells possess tumor initiating potential. Recent data from both hematologic malignancies and solid tumors have suggested that there are only minor populations of cells in each malignancy that are capable of tumor initiation which are the cancer stem cells (CSC) [4], [5], [6], [7], [8], [9], [10], [11], [12], [13], [14], [15]. These cells appear to be capable of asymmetric division and self-renewal, and are only a minor fraction among the bulk of more differentiated cells in the tumor [16], [17], [18].

Recently, CSCs have been studied in different primary tumor types in order to develop CSC-specific therapies [6], [11], [19], [20], [21]. Interestingly, certain tumors are highly resistant to chemotherapy and other forms of treatment and although aggressive treatments destroy the majority of the cancerous cells, a small fraction of the cells survive and often regenerate into even larger masses of tumor cells [22], [23], [24]. Effective therapies for cancer patients require a thorough understanding of mechanisms leading to tumor development and drug resistance.

The recent discovery of CSCs has played a pivotal role in changing our view of carcinogenesis and chemotherapy. CSCs are thought to be responsible for the formation and growth of neoplastic tissue. The CSCs are naturally resistant to most current chemotherapy due to their quiescent nature. This may explain why traditional chemotherapies can initially reduce the majority of the tumor bulk but fail to eradicate it in full, allowing eventual recurrence [1], [11], [17], [18], [22]. CSCs are more resistant to therapy not only secondary to quiescence, but also due to increased expression of anti-apoptotic proteins and drug efflux transporters. Cancer treatments available today mostly exploit the proliferative and metastatic potentials of the cancer cells; therefore, the majority of treatments are targeted at rapidly dividing cells and at molecular targets that represent the bulk of the tumor. This may explain the failure of treatments to eradicate the disease or to prevent recurrence of cancer. Additionally, if a drug affects the growth of only a minor population of cells, there will be only a minimal decrease in the growth of the tumor in the short term.

Theoretically, identification and characterization of the CSCs may allow the development of treatment modalities that target the cancer stem cells rather than the rapidly dividing cells in the cancer.

Prolonged exposure of humans and experimental animals to the altered gravitational conditions of space flight has adverse effects on different cellular systems. The effects of hypogravity environment on tumor growth and carcinogenesis are yet unknown and this research field still lacks systematic investigation on hypogravity induced gene expression, which is the key information needed to ultimately unfold the mechanism behind hypogravity-induced diseases. To this end, we have studied the effects of simulated hypogravity on human osteosarcoma cells cultured in the NASA-developed hydrofocusing bioreactor (HFB) and in the rotary cell culture system (RCCS). Various designs of rotating-wall vessels have been used to create a three-dimensional culture environment with variable shear stress and hypogravity simulating that in space [25]. The HFB (Celdyne, Houston, TX) developed by NASA at the Johnson Space Center, is a fluid filled dome, which rotates at a specified speed and has a conical spinner to provide a unique hydrofocusing capability that will allow for an extremely low-shear culture environment and a nylon gas transfer membrane [25]. The RCCS consists of a horizontally rotated culture vessel oxygenated by a flat silicone rubber gas transfer membrane [26]. The NASA-designed HFB bioreactor and the RCCS have been successfully used on Earth and in Space to enable investigators to use modeled or actual hypogravity, respectively, to study the role of gravity on the formation of three-dimensional mammalian cell tissue models and production of bio-products [25], [27], [28], [29], [30], [31].

In this study we show that cancer stem cells are stimulated to proliferate when they are cultured in the hypogravity conditions produced by the HFB and that the reduction of gravity simulated in the HFB sensitizes CSCs to chemotherapeutic agents.

## Results

### Osteosarcoma stem-like cells proliferate in the Hydro-Focusing Bioreactor (HFB), but not in rotary cell culture system (RCCS)

Modeled hypogravity is a condition in which cells are in constant free fall and in which they are able to grow in an anchorage independent manner. Since the effects of hypogravity on tumors are unknown, we thought to investigate the effects of modeled hypogravity in the absence of shearing stress on the growth capacity of tumor cells of different embryonic origin. We have used an HFB developed by NASA at the Johnson Space Center, which is composed of a 50 mL fluid filled dome that rotates at a specified speed and that has an internal conical spinner that hydro-focuses the cell culture allowing for a low-shear environment.

**Figure 1:**
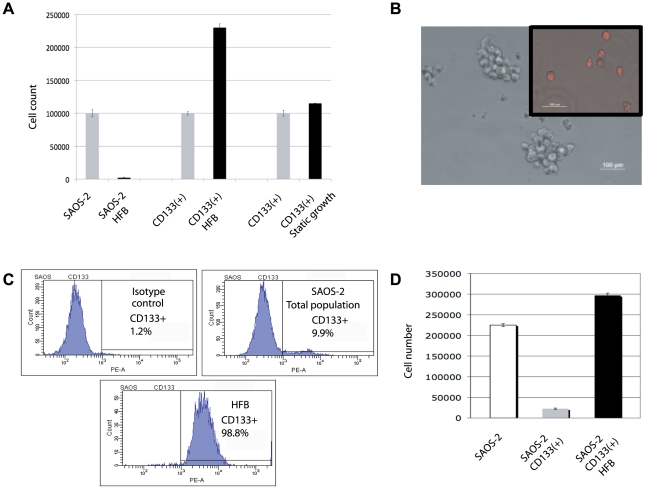
CD133(+) SAOS-2 cells are selected in the hydrofocusing bioreactor. A) Trypan blue exclusion cell count of SAOS-2 cells (osteosarcoma cells) and SAOS-2 cells cultured in the HFB, CD133(+) SAOS-2 cells and CD133(+) SAOS-2 cells cultured in the HFB, and of CD133(+) SAOS-2 cells and CD133(+) SAOS-2 cells cultured in normal gravity condition. Gray bars indicate the number of cells placed in the control on day-1. Black bars indicate the number of viable cells recovered after 5 days of culture in that culturing condition. B) Bright field contrast phase image (20×) of 3-D culture (spheres) formed from SAOS-2 cells that were grown in the HFB for 5 days. Inset (top right) shows an image of CD133 immunofluorescence staining of SAOS-2 cells grown in the bioreactor for 5-days. C) Flow cytometry chart of the CD133 immunophenotype of SAOS-2 cells. An isotype antibody was used as a control. D) Trypan blue exclusion cell counts of an optimized experiment of HFB culture of SAOS-2 cells in the HFB after seven days. CD133(+) SAOS-2 cells cultured in the bioreactor were selected and proliferated 15-fold after seven days. White bar indicates the number of SAOS-2 cells seeded in the HFB. Black bar indicates the number of viable CD133(+) SAOS-2 cells recovered after 7 days of optimized culture in the bioreactor. Gray bar indicate the number of CD133(+) cells present in the parental SAOS-2 population. The data is representative of three separate experiments yielding comparable results.

To our surprise, we found that when a known number of SAOS-2 cells were seeded in the HFB, only a small fraction of these cells survived the treatment, independently from the cellular density seeded in the bioreactor. **Figure 1A** shows that the number of viable SAOS-2 cells cultured for 5-days in the HFB was dramatically reduced.

Interestingly, SAOS-2 cells cultured in the bioreactor formed cell spheres and appeared to be significantly smaller than SAOS-2 cells cultured in dishes and harvested by trypsin treatment (data not shown). **Figure 1B** shows a 20× bright field picture of spheres formed from SAOS-2 cells that were grown in the HFB for 5 days. Cells were removed from the HFB reactor and pictures were taken immediately using an inverted microscope using contrast phase.

Because of their apparent change in morphology and ability to form spheres in the HFB environment and because others have demonstrated the presence of CD133(+) cells within primary bone sarcomas, as well as the osteosarcoma cell lines MG-63, OS-521, 0S-01-187, OS-99-01, and SAOS-2 and the chondrosarcoma cell line CS-828 [32], [33], we hypothesized that the HFB selected cells were CD133(+). To test this hypothesis we immunophenotyped these cells with an antibody directed against CD133 (Miltenyi, Germany), and found that HFB selected cells were 100% positive to CD133, a typical stem cell marker of mesenchymal origin (**Figure 1B** **, inset panel and** **Figure 1C**). Since the SAOS-2 cells recovered from the bioreactor were all CD133(+) (98.8%) (**Figure 1C**), we wanted to determine whether the CD133(+) cells not only survived but also proliferated in the hypogravity environment. To this end, we selected CD133(+) cells from the SAOS-2 or HOS cell lines using a MACSorting system and challenged these CD133(+) cells to a 5-day run in the HFB. Interestingly, SAOS-2 or HOS cells MACSorted with an antibody against CD133 and cultured in the bioreactor for 5 days increased in number by two-fold (**Figure 1A**). **Figure 1A** shows the trypan blue exclusion cell counts of CD133(+) SAOS-2 viable cells recovered after 5 days of culture in the bioreactor.

After a few trials of cell culture optimization, we were able to achieve a 15-fold increase in proliferation of CD133(+) cancer stem-like cells from the parental SAOS-2 cells over a seven-day period by stopping the reactor every 24-hours and gently mixing the culture 10 times in an orthogonal manner over a period of one minute, which allowed for redistribution of the media in the dome and mixing of the nutrient with the proliferating cells (**Figure 1D**). Figure 1D shows that following an optimized protocol of cell culture in the HFB, it is possible to select and to proliferate a specific population contained in the SAOS-2 parental cell line. Similar results were achieved using various other cancer cell lines of different tissue origin and which express a variable amount of the CD133 marker (HOS, U2OS, T98G, U87MG, Du145, LNCap, WI38, H23, Hep3b, Hela, Mewo, HO-1 cells, HN12 and HN30), see **Table 1**, and data not shown.

**Table 1:** Percentage of positivity ± standard deviation to CD133 of the various cell lines tested.

Cell Line	Tumor type	% CD133 (+)	%CD34(+)	%CD38(+)
HOS	Human osteosarcoma	37.7±7.8	5.58±2.2	0.52±0.07
SAOS-2	Human osteosarcoma	10.86±6.8	1.14±0.13	4.08±0.08
U2OS	Human osteosarcoma	1.06±0.05	70.5±0.6	0.51±0.06
T98G	Human glioblastoma	0.3±0.1	0.4±0.2	0.2±0.01
U87MG	Human glioblastoma	0.2±0.1	0.8±0.3	0.4±0.2
Du145	Human prostate adenocarcinoma	0.6±0.3	0.3±0.05	1.2±0.03
LNCap	Human prostate adenocarcinoma	1±0.6	1.9±0.3	0.4±0.2
WI38	Human lung fibroblast	2.4±1	1.6±0.5	0.5±0.2
H23	Human lung adenocarcinoma	5.36±0.75	5.18±1.1	0.4±0.13
Hep3b	Human hepatocarcinoma	96.8±0.6	0.6±0.03	0.1±0.01
Hela	Human cervical carcinoma	0.8±0.7	2.5±1	0
Mewo	Human melanoma	0.5±0.1	0	0
HO-1	Human melanoma	1.4±0.6	0.8±0.2	0.3±0.1
HN-12	Human H&N squamo cellular carcinoma	0.4±0.3	0.2±0.1	0.3±0.1
HN-30	Human H&N squamo cellular carcinoma	0.1±0.1	0.2±0.1	0.2±0.1

Another bioreactor that simulates hypogravity, the 50 mL rotary cell culture system (RCCS) [26] manufactured by Synthecon (Houston, TX), was used to compare results generated using the HFB. In a parallel experiment in which we seeded an equal number of SAOS-2 cells in the same tissue culture incubator with identical conditions of rotor speed, CO2, temperature, and vessel volume (50 mL), the HFB increased CD133(+) cell growth from SAOS-2 cells compared to the RCCS vessel and to earth gravity control. 5×10∧6 SAOS-2 cells were seeded in the HFB or RCCS bioreactors of which 350,000 (7%) were CD133(+) and 4,650,000 CD133(−) (**Table 2**). Culture media, oxygenation, speed, temperature and CO_2_ were kept consistently constant for the two culture systems and in the same incubator for a 7-days run. After a 7-day run, the vessels were harvested and cells were reacted with an antibody fluoresceinated against CD133 (Miltenyi, Germany) and counted using a BD Facs Aria flow cytometer. An isotype antibody was used as control. From the comparison of the two bioreactor cultures, we showed that a (+)15-fold increase in CD133(+) cells number (5,370,960 CD133+ cells) was achieved using the HFB on a 7-day run. No proliferation of CD133(+) cells was observed in the culture derived from the RCCS culture vessel. The RCCS culture vessel showed instead a drastic reduction in the CD133(+) cell population [(−)4.8 fold decrease] from the number of CD133(+) cells seeded in the bioreactors on day-0 (**Table 2**). In fact, the number of SAOS-2 CD133(+) cells decreased from 350,000 to 72,800 in a 7-day run in the RCCS, while the HFB grown SAOS-2 CD133(+) cells increased from 350,000 to 5,370,960 during an equivalent period of time.

**Table 2:** Comparison of CD133(+) and (−) cel counts ± standard deviation of SAOS-2 cells grown in the HFB or RCCS vessels.

Device	Total cell Input	CD133(+) cell Input	Total cell Output	CD133(+) cell Output	Fold Change
**RCCS**	5,000,000	350,000	5,200,000	72,800	(**−**) 4.8
**HFB**	5,000,000	350,000	5,370,960	5,370,960	(**+**) 15.3

Because of this dramatic difference in the growth of SAOS-2 CD133(+) cells in the two bioreactors generating hypogravity (HFB and RCCS), we wanted to verify if pH could be a variable causing the effects on cell growth observed. Therefore, we measured the pH of the media of the HFB and RCCS bioreactors kept in the same incubator with a CO_2_ level set at 5% and a constant temperature of 37°C before and after a run of 7-days. We compared the pH of the media from the HFB and RCCS vessels with the conditioned media of control cells grown in a Petri dish in earth gravity condition versus unspent D-MEM medium. No statistically significant difference was found among the different cell culture conditions by repeating the experiments three times. In fact, unspent medium showed a pH of 7.82±0.04. Medium from control SAOS-2 cells grown in a Petri dish in normal gravity conditions showed a pH of 7.96±0.23; medium from SAOS-2 cells grown in the HFB had a pH of 7.71±0.23; and medium from SAOS-2 cells grown in the RCCS bioreactor showed a pH of 7.9±0.11, demonstrating that the different growth of CD133(+) SAOS-2 cells observed between the RCCS and HFB vessels was not due to changes in pH in the culturing media.

### Osteosarcoma cells die by apoptosis in the Hydro-Focusing Bioreactor (HFB)

Since only a small fraction of the osteosarcoma SAOS-2 as well as HOS cells placed in the HFB cell culture system were recovered after 3 or 5 days of culture, we tested whether these cells were being eliminated from the initial population by causing them to undergo apoptosis. Therefore, we compared the rates of apoptosis of SAOS-2 cells cultured in the HFB for 3 and 5 days to MACSorted CD133(+) cells cultured in the HFB for 3 and 5 days.

**Figure 2:**
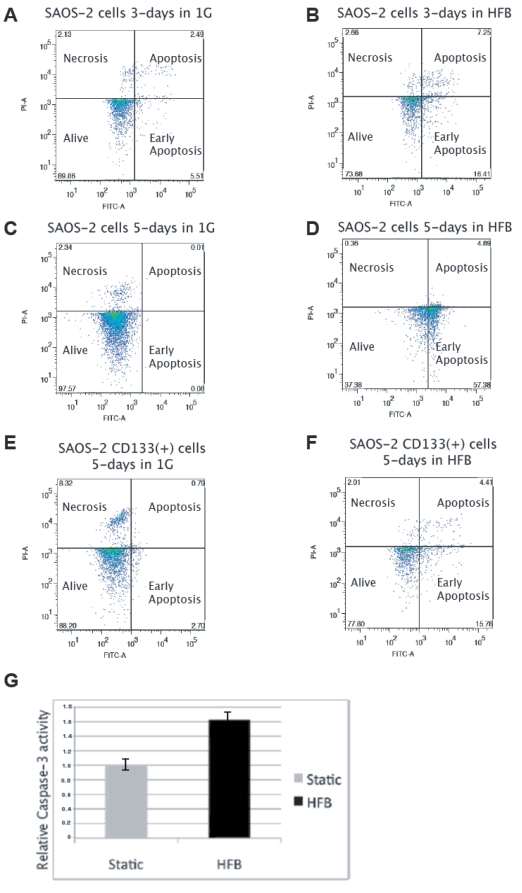
SAOS-2 cells die by apoptosis after 5 days of culture in the hydrofocusing bioreactor as evidenced by Annexin-V staining/Facs analysis and caspases-3 colorimetric assay. **A**) Annexin-V staining of SAOS-2 cells cultured in 1-G for 3 days. The analysis allows to distinguish in the diagram between living cells (lower left quadrant), early apoptotic cells (lower right quadrant), apoptotic cells (upper right quadrant), and necrotic cells (upper left quadrant). **B**) Annexin-V staining of SAOS-2 cells cultured in the HFB for 3 days. **C**) Annexin-V staining of SAOS-2 cells cultured in 1-G for 5 days. **D**) Annexin-V staining of SAOS-2 cells cultured in the HFB for 5 days. **E**) Annexin-V staining of CD133(+) MACSorted SAOS-2 cells which were cultured in 1-G for 5 days. **F**) Annexin-V staining of CD133(+) MACSorted SAOS-2 cells which were cultured in the HFB for 5 days. **G**) Relative caspase-3 activity of SAOS-2 cells (total population) cultured for 5 days in the HFB compared to SAOS-2 cells (total population) grown in static 1G condition.

To this end we have analyzed SAOS-2 cells cultured for 3-days or 5-days in the bioreactor and found that SAOS-2 cells cultured in the HFB for 3 or 5-days die by apoptosis as shown by a flow cytometric assay of Annexin-V and propidium iodide staining of the samples (**Figures 2 B and D**). 24% of the cells were found in apoptosis after 3-days of culture in the HFB. Interestingly, the fraction of cells dying by apoptosis increased to 62% after 5-days of culture in the HFB, confirming the initial observation (**Figure 1**). Most importantly, samples of CD133(+) MACSorted cells grown in the HFB for 5-days didn't show such an increase in apoptosis by Annexin V staining when compared to the control sample (**Figure 2E and F**). In the same culture conditions in the HFB, the CD133(+) cells proliferate while the CD133(−) cells instead die by apoptosis.

We have also investigated this phenomenon by measuring the activity of caspases by using a colorimetric caspases kit that measures the activity of caspases-3 in the samples. According to the data presented we found that SAOS-2 cells (total population) cultured in the HFB for 5 days showed an increase of 1.6-fold in the caspases-3 activity detected (**Figure 2 G**), which is a measure of the apoptotic index in these cells.

### Stem cell marker expression increases following culture in the Hydro-Focusing Bioreactor

**Figure 3:**
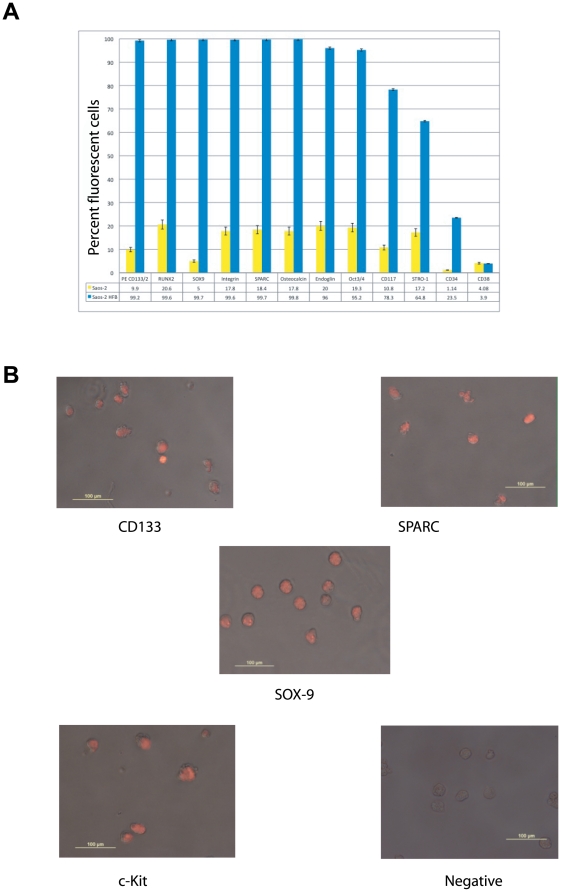
Fluorescence index of various stem cell and differentiation markers examined in cells cultured in 1-G static condition, sorted by CD133 antibody or not, and after culture in the bioreactor for 5 days. **A**) Diagram of the expression levels of various bio-markers. Lavender bars indicate the percentage of cells expressing basal levels of the various markers in parental SAOS-2 cells cultured in normal 1-G gravity condition (control). Purple bars indicate the percentage of cells expressing the various markers in CD133(+) MACSorted SAOS-2 cells that were isolated from parental SAOS-2 cells cultured in normal 1-G gravity condition. Yellow bars indicate the percentage of cells expressing the various markers in CD133(+) MACSorted SAOS-2 cells that were cultured in hypogravity condition. Light blue bars indicate the percentage of cells expressing the various markers in parental SAOS-2 cells that were cultured in hypogravity condition for 5-days, resulting in a population of selected and proliferated CD133(+) SAOS-2 cells. **B**) Immunofluorescence staining (40×) and positivity of SAOS-2 cells to CD133, Sox-9, SPARC, and CD117/c-Kit following growth in the bioreactor for 5-days.

Perhaps the most intriguing capability conferred by the rotating, low-shear bioreactor system is the opportunity to study, under controlled conditions, the interaction of cells of a given type or the interaction of one cell type with another when cells in suspension are free to form their own associations. We wanted to analyze the expression levels of various markers related to the development of stem cells of mesenchymal origin. The expression of CD133, CD34, CD38, Osteocalcin, Sparc, Sox-9, RunX-2, Stro-1, CD117/c-Kit, Oct3/4, Endoglin, and Integrin-ß1 was examined by flow cytometry. **Figure 3A** shows the percent of fluorescent cells of the various markers examined in SAOS-2 cells cultured in static condition and after culture in the HFB for 5-days. The expression levels and the number of cells expressing the examined markers increased after 5 days of culture in the HFB vessel compared to the cells cultured in normal gravity conditions. Interestingly, the SAOS-2 cells grown in the HFB vessel showed the highest relative increase in the number of cells positive to Sox-9, CD133, Osteocalcin, Integrin-ß1, Sparc, RunX-2, and Endoglin (94.7%, 89.3%, 82%, 81.8%, 81.3%, 79%, and 76%, respectively), when compared to SAOS-2 cells cultured under a normal gravity conditions. Oct3/4, CD117, Stro-1, and CD34 also showed an increase in the number of cell number positivity (75.9%, 67.5%, 47.6%, 22.36%, respectively) comparing 1G-growth versus SAOS-2 cells grown in simulated hypogravity for 5-days. We found no change in the number of CD38 positive cells in the same conditions. The experiment was repeated 5 times with comparable results and standard deviations were calculated and are reported on the diagram as error bars. **Figure 3B** shows an example of immunofluorescence staining and positivity of SAOS-2 cells to CD133, Sox-9, Sparc, and CD117/c-Kit following growth in the HFB vessel for 5 days.

### CD133(+) cells grow three-dimensionally in the Hydro-Focusing Bioreactor and form spheres

**Figure 4:**
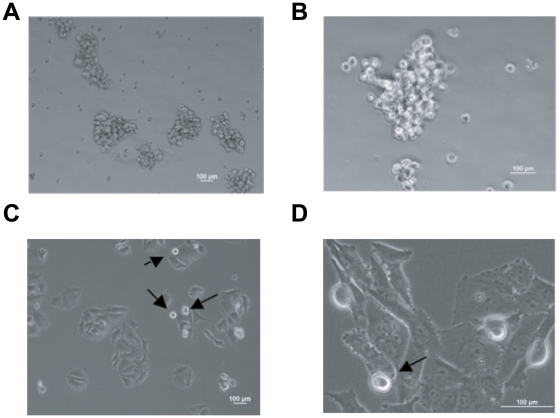
Phase contrast images of spheres formed by HFB SAOS-2 CD133(+) enriched osteosarcoma cells. **A**) HFB grown SAOS-2 osteosarcoma cells (CD133+) are able to proliferate and assemble three-dimensionally as sarcosheres after 3-days of culture in the bioreactor (10× magnification). **B**) HFB grown SAOS-2 osteosarcoma cells (CD133+) are able to proliferate and assemble three-dimensionally as sarcosheres after 3-days of culture in the bioreactor (40× magnification). **C**) HFB grown SAOS-2 osteosarcoma cells (CD133+) seeded into an irradiated plastic culture dish, reconstitute the normal attached phenotype of the parental SAOS-2 cells after one week of culture (10× magnification). Arrows point at CD133(+) SAOS-2 cells showing a rounded and weakly attaching phenotype. **D**) HFB grown SAOS-2 osteosarcoma cells (CD133+) seeded into an irradiated plastic culture dish, reconstitute the normal attached phenotype of the parental SAOS-2 cells after one week of culture (40× magnification). Arrow points at CD133(+) SAOS-2 cells showing a rounded and weakly attaching phenotype.

The SAOS-2 CD133(+) enriched osteosarcoma cells proliferate and assemble three-dimensionally as sarcospheres after 3-days culture in the bioreactor, which is yet another characteristic of stem cell growth. **Figures 4 A and B** show at 10 and 40× magnification power, respectively, the sarcospheres grown in the bioreactor after 3 days of culture in simulated hypogravity. Interestingly, the CD133(+) SAOS-2 cells that were grown in simulated hypogravity were able to re-establish the parental cell line (constituted by about 10%±6.8 CD133 (+) cells and 90%±6.8 CD133(−) cells) after a period of one week when seeded into treated culture dishes, which allow for adherent cells to attach to the plastic environment. **Figure 4 C and D** (10× and 40×, respectively) show the SAOS-2 CD133(+) cells proliferated and selected with the HFB that were subsequently grown in attaching tissue culture dishes. In the reconstituted cell culture some not-adhering cells were noticeable; these could be stem-like cells also known as cancer stem cells.

**Figure 5:**
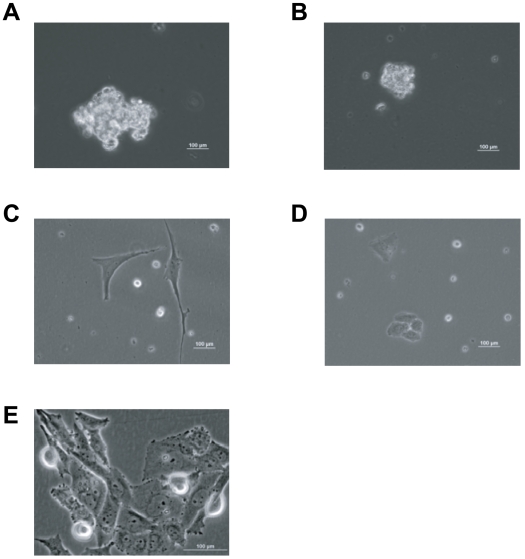
Phase contrast images of spheres formed by MACSorted CD133(+) SAOS-2 osteosarcoma cells in ultra low-attaching dishes. **A**) MACSorted CD133(+) SAOS-2 cells assemble three-dimensionally as sarcosheres after 2 weeks of culture in ultra low-attaching dishes (20× magnification). **B**) Another example of MACSorted CD133(+) SAOS-2 cells forming sarco-spheres after 2 weeks of culture in ultra low-attaching dishes (10× magnification). **C**) SAOS-2 osteosarcoma cells deriving from three-dimensional cultures grown in ultra low-attaching dishes seeded into attaching dishes show an adherent a differentiated phenotype after 3-days. **D**) SAOS-2 osteosarcoma cells deriving from three-dimensional cultures grown in ultra low-attaching dishes seeded into attaching dishes show an adherent a differentiated phenotype after 5-days. **E**) SAOS-2 osteosarcoma cells deriving from three-dimensional cultures grown in ultra low-attaching dishes seeded into attaching dishes show an adherent and differentiated phenotype after 10 days.

It is known that stem-like cells will form cell spheres when cultured in ultra low-attaching dishes. We therefore tested the ability of CD133(+) SAOS-2 MAC-sorted cells to form cell clusters if placed in ultra low-attaching dishes. Their ability to form clusters was less efficient when compared to those cells that have been cultured in the hydrofocusing bioreactor, but they were still able to form spheres. **Figure 5 A and B** show spheres formed by SAOS-2 CD133(+) cells in ultra low-attaching dishes. We also tested the ability of the SAOS-2 CD133(+) MACSorted and enriched cells to attach to tissue culture dishes and to reconstitute the parental SAOS-2 cell line. As expected, the MACSorted CD133(+) enriched cells placed in attaching tissue culture dishes after being cultured in ultra low-attaching dishes for two weeks, reconstituted the parental SAOS-2 cell line after one week of culture by attaching to the dish, manifesting a flattened and differentiated phenotype over time. **Figure 5C and D** shows adherent SAOS-2 cells derived from CD133(+) MACSorted cells grown in adherent tissue culture dishes. **Figure 5 E** shows the SAOS-2 cell line reconstituted after 10-days of inoculation of CD133(+) enriched cells in adhering tissue culture dishes.

**Figure 6:**
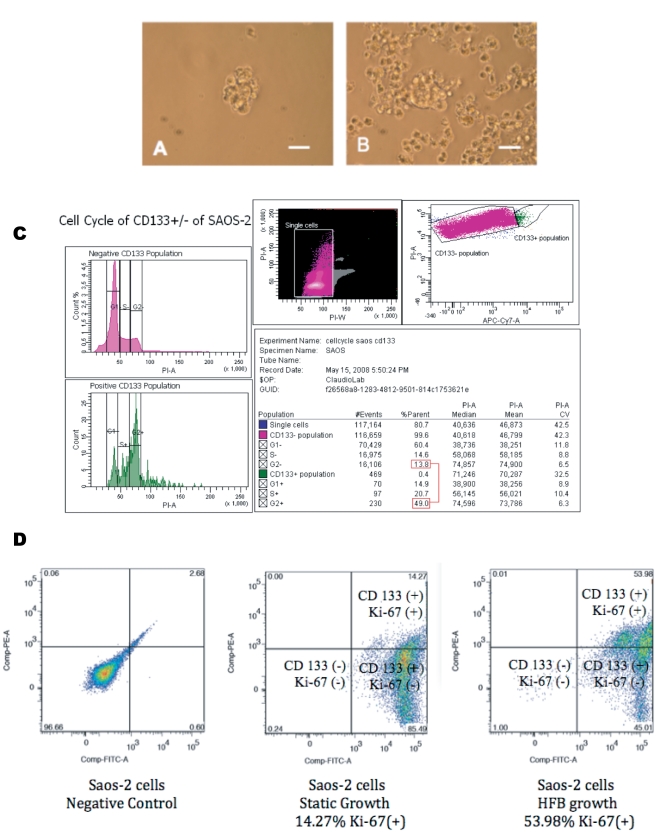
The SAOS-2 cell line is composed of two distinct populations. **A**) Contrast phase microscopy of a soft agar assay of parental SAOS-2 cells. **B**) Contrast phase microscopy of a soft agar assay of HFB CD133(+) proliferated SAOS-2 cells. **C**) Propidium iodide flow cytometry analysis of SAOS-2 cells grown in 1-G culture sorted by their CD133 status showing two distinct populations: the CD133(+) and CD133(−) cells with a different cell cycle profile. D) Immunophenotype of SAOS-2 cells grown in 1-G versus HFB culture showing that the SAOS-2 cells grown for 5 days in simulated hypogravity contain the majority of the cells stained for Ki-67 and therefore in the S-phase of the cell cycle.

An additional indication that two distinct populations are identifiable in the SAOS-2 cell line is the evidence that CD133(+) MACSorted SAOS-2 cells have enhanced ability to grow in soft agar when compared to the parental cells. The cells were plated in 6-well plates performing 6 replicates of each experimental point. Equal number of SAOS-2 cells and of CD133(+) SAOS-2 cells (5×10^3^ cells/well) were seeded in 6 replicas into a six-well dish. The efficiency of CD133(+) SAOS-2 cells to grow in soft agar and to assemble in three-dimensional structures is much higher than that of the parental SAOS-2 cells. Six hundred and forty ±240 colonies were counted from the soft-agar 6-well dishes seeded with the CD133(+) SAOS-2 enriched cells compared to only 20±12 colonies counted from the soft-agar dishes seeded with the CD133(−) SAOS-2 cells. Interestingly, 120±80 colonies were counted from the soft-agar dishes seeded with the parental SAOS-2 cells, suggesting that the ability of forming colonies in soft-agar could be due to the presence of a fraction (consistently, about 10%±6.8) of stem-like cells within the SAOS-2 cell line we have in our laboratory (**Table 3**). **Figure 6** shows an example of a soft-agar assay performed with the parental tumor cell line (**Figure 6A**) or with the CD133 (+) enriched fraction of SAOS-2 cells (**Figure 6B**). The parental SAOS-2 cells formed spheres, but with very low efficiency when compared to the enriched CD133 (+) cells.

**Table 3:** Average number of colonies counted ± standard deviation in 6-well dishes in a triplicate experiment.

	Parental SAOS-2	CD133(+)	CD133(−)
Total number of colonies	120±80	640±240	20±12

Further evidence that there are two distinct populations in the SAOS-2 cell line, which appears to be composed of CD133(+) and (−) cells, is that these two different populations have a distinct cell cycle profile. We performed a flow cytometry analysis of SAOS-2 cells grown in 1G culture and found that SAOS-2 CD133(+) cells, which were sorted by their CD133 status and stained with propidium iodide, had a different cell cycle profile with respect to the CD133(+) population. **Figure 6C** shows a representative flow cytometric assay of SAOS-2 cells demonstrating that the CD133(−) SAOS-2 population had 13.8% of cells in the G2/M phase of the cell cycle, while the CD133(+) cells showed an increased fraction (49%) of cells in the G2/M phase of the cell cycle. Comparable results were obtained in parallel and repeated experiments.

Interestingly, SAOS-2 cells grown in the bioreactor for 5 days and stained with an anti-CD133 (Miltenyi, Germany) and the proliferation marker anti-Ki-67 demonstrated increased proliferation of the cells compared to the SAOS-2 cells grown in 1G cultures. **Figure 6D** shows a representative flow cytometry assay of SAOS-2 cells demonstrating that the cells cultured in the HFB proliferate at a different rate than the total SAOS-2 cell population. In fact, the fraction of SAOS-2 CD133(+) cells, which are also positive to Ki-67, increased from 14.27% to 53.98% comparing cells cultured in 1G-growth versus those cells cultured for 5 days in HFB simulated hypogravity condition, respectively. Comparable results were obtained in parallel and repeated experiments.

### Simulated hypogravity enhances apoptosis and sensitizes cancer stem cells to chemotherapy

CSCs are thought to be responsible for initiating and maintaining the disease. Some types of tumors are highly resistant to chemotherapy and other forms of treatment. Although aggressive treatments can destroy the majority of the cancerous cells, a small fraction of them remain and often later regenerate into even larger masses of tumor cells that are even more treatment resistant [17], [18], [20]. Our recent discovery that cancer stem cells are stimulated to proliferate when they are placed in hypogravity conditions encourages the idea to test the ability of simulated hypogravity to sensitize tumor stem cells to chemotherapeutic agents.

**Figure 7:**
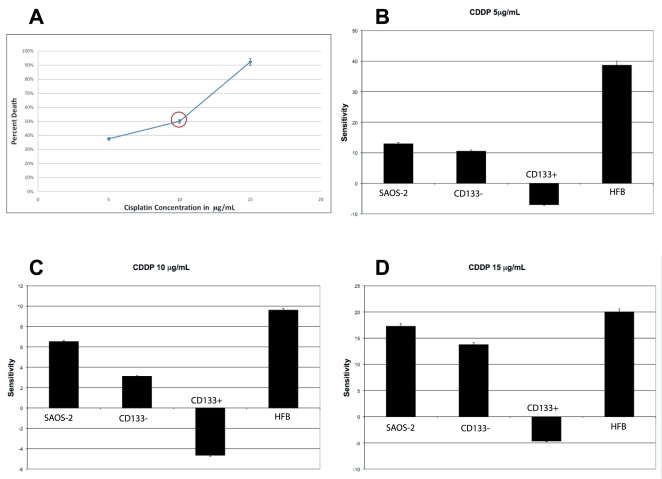
Sensitivity of SAOS-2 cells to cisplatin following growth in simulated hypogravity. **A**) LD_50_ for cisplatin determined for the SAOS-2 cells. An LD_50_ of 10µg/mL for cisplatin was determined exposing the SAOS-2 cells to a 24-hours treatment using the MTT assay. Comparable results were obtained with cell count by trypan blue exclusion. **B**) Histogram showing the sensitivity of SAOS-2 cells to 5µg/mL of cisplatin following a 24-hours treatment, using an MTT assay. CD133(+) cells are resistant to the chemotherapy treatment, but the CD133(+) SAOS-2 cells proliferated and selected with the HFB culture system are sensitive, instead. **C**) Histogram showing the sensitivity of SAOS-2 cells to a clinically relevant dose of 10µg/mL of cisplatin following a 24-hours treatment, using an MTT assay. CD133(+) cells are resistant to the chemotherapy treatment, but the CD133(+) SAOS-2 cells proliferated and selected with the HFB culture system are sensitive, instead. **D**) Histogram showing the sensitivity of SAOS-2 cells of 15µg/mL of cisplatin following a 24-hours treatment, using an MTT assay. CD133(+) cells are resistant to the chemotherapy treatment, but the CD133(+) SAOS-2 cells proliferated and selected with the HFB culture system are sensitive, instead.

We have therefore tested the ability of hypogravity to sensitize the osteosarcoma cells to various chemotherapy agents used in cancer therapy. We have discovered that the HFB-hypogravity environment sensitized the CD133(+) resistant osteosarcoma cells to various chemotherapy agents at clinically relevant doses. We have tested the sensitivity of osteosarcoma cells to cisplatin, doxorubicin and methotrexate, which are the drugs of choice for this tumor type in the clinical settings. SAOS-2 cells were grown for 5-days in the HFB and then harvested. 1×10^4^ cells were plated in 96-well dishes and were subjected, along with a comparable number of cells of the other samples, to 5, 10, and 15 µg/mL of cisplatin for 24 hours and then subjected to MTT assay (**Figure 7**). The same numbers of cells were used for the doxorubicin and methotrexate treatments. For methotrexate treatments, the cells were subjected to 4, 11, 22, and 45 µg/mL of methotrexate for 24 hours and then subjected to MTT assay. For doxorubicin treatments the samples were treated with 0.5, 1.1, and 2.2 µg/mL of doxorubicin for 24 hours and then subjected to MTT assay. The MTT assay is a convenient colorimetric assay of mitochondrial viability that assesses the number of viable cells versus the number of dead cell in a given sample. **Figures 7** **,** **8** **, and** **9** show that the CD133(+) cells grown in the HFB increased their sensitivity to the cisplatin, doxorubicin, and methotrexate treatments, respectively. These phenomena may be due to the fact that the cancer stem cells were stimulated to proliferate by culture in the HFB environment. The CD133(+) cells that were not subjected to the hypogravity environment appear to be resistant to the same treatment regiment. Similar results were achieved by treating the cells with the chemotherapeutic agents for 1 hour and subjecting the samples to an MTT assay 24 hours later (data not shown). These data have been confirmed with other techniques, such as measurement of cell viability by trypan blue exclusion cell-count before and after chemo treatment, as well as by flow-cytometry analysis (data not shown).

**Figure 8:**
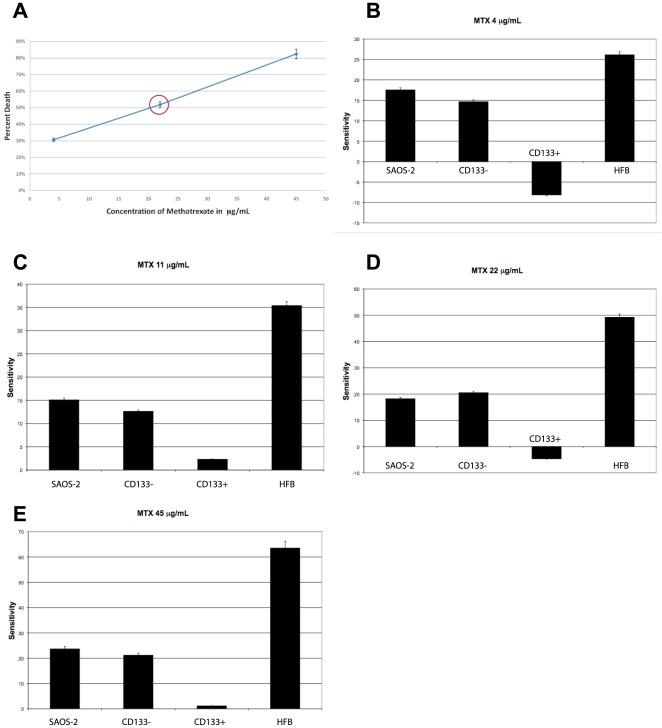
Sensitivity of SAOS-2 cells to methotrexate following growth in simulated hypogravity. **A**) LD_50_ for methotrexate determined for the SAOS-2 cells. An LD_50_ of 22µg/mL for methotrexate was determined exposing the SAOS-2 cells to a 24-hours treatment to the drug, using the MTT assay. Comparable results were obtained with cell count by trypan blue exclusion. **B**) Histogram showing the sensitivity of SAOS-2 cells to 4µg/mL of methotrexate following a 24-hours treatment, using an MTT assay. CD133(+) cells are resistant to the chemotherapy treatment, but the CD133(+) SAOS-2 cells proliferated and selected with the HFB culture system are sensitive, instead. **C**) Histogram showing the sensitivity of SAOS-2 cells to 11µg/mL of methotrexate following a 24-hours treatment, using an MTT assay. CD133(+) cells are resistant to the chemotherapy treatment, but the CD133(+) SAOS-2 cells proliferated and selected with the HFB culture system are greatly sensitive, instead. **D**) Histogram showing the sensitivity of SAOS-2 cells to a clinically relevant dose of 22µg/mL of methotrexate following a 24-hours treatment, using an MTT assay. CD133(+) cells are resistant to the chemotherapy treatment, but the CD133(+) SAOS-2 cells proliferated and selected with the HFB culture system are sensitive, instead. **E**) Histogram showing the sensitivity of SAOS-2 cells to 45µg/mL of methotrexate following a 24-hours treatment, using an MTT assay. CD133(+) cells are resistant to the chemotherapy treatment, but the CD133(+) SAOS-2 cells proliferated and selected with the HFB culture system are sensitive, instead.

**Figure 9:**
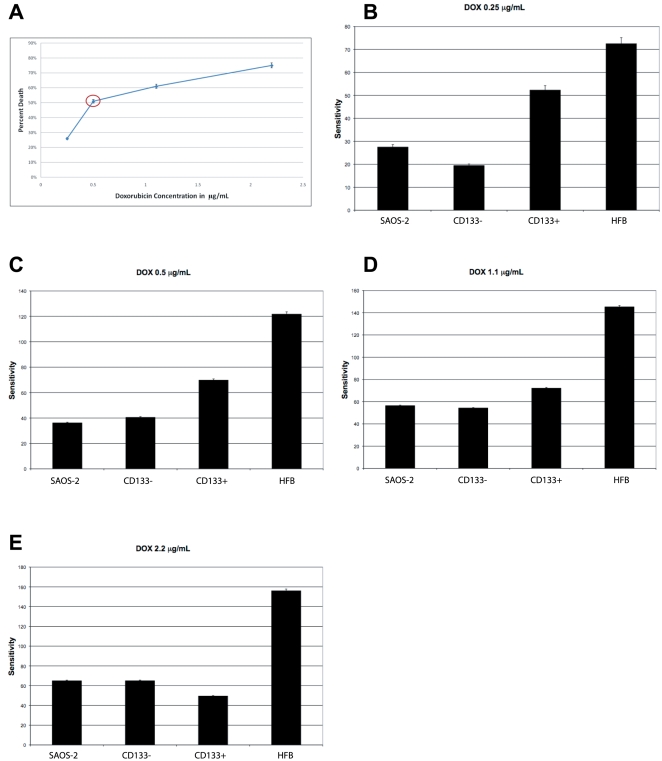
Sensitivity of SAOS-2 cells to doxorubicin following growth in simulated hypogravity. **A**) LD_50_ for doxorubicin determined for the SAOS-2 cells. An LD_50_ of 0.5µg/mL for doxorubicin was determined exposing the SAOS-2 cells to a 24-hours treatment to the drug, using an MTT assay. Comparable results were obtained with cell count by trypan blue exclusion. **B**) Histogram showing the sensitivity of SAOS-2 cells to 0.25µg/mL of doxorubicin following a 24-hours treatment, and using an MTT assay. CD133(+) cells are sensitive to the chemotherapy treatment, but the CD133(+) SAOS-2 cells proliferated and selected with the HFB culture system are even more sensitive to the treatment, instead. **C**) Histogram showing the sensitivity of SAOS-2 cells to 0.5µg/mL of doxorubicin following a 24-hours treatment, using an MTT assay. CD133(+) cells show sensitivity to the clinically relevant dose of 0.5 µg/mL of doxorubicin, however the CD133(+) SAOS-2 cells proliferated and selected with the HFB culture system are greatly sensitized to the chemotherapy treatment. **D**) Histogram showing the sensitivity of SAOS-2 cells to a dose of 1.1µg/mL of doxorubicin following a 24-hours treatment, and using an MTT assay. The CD133(+) cells show sensitivity to a dose of 1.1µg/mL of doxorubicin, however the CD133(+) SAOS-2 cells proliferated and selected with the HFB culture system are sensitized to the chemotherapy treatment. **E**) Histogram showing the sensitivity of SAOS-2 cells to 2.2µg/mL of doxorubicin following a 24-hours treatment, and using an MTT assay. CD133(+) cells are sensitive to the chemotherapy treatment, but the CD133(+) SAOS-2 cells proliferated and selected with the HFB culture system are sensitive, instead.

Interestingly, the CD133(+) osteosarcoma cells had an overall resistance to any dose of the cisplatin and methotrexate treatments tested, whether above or below the assessed LD_50_. However, the cancer cells stimulated to grow in the bioreactor, which are CD133(+), showed enhanced cell death even at doses well-below the LD_50_, suggesting that environments in which lack of gravity could be simulated may be used to lower the necessary dose of chemo treatments for patients.

Surprisingly, the CD133(+) osteosarcoma cells were sensitive to treatments with doxorubicin even at doses lower than the LD_50_. However, the CD133(+) cancer cells stimulated to grow in the bioreactor showed enhanced cell death also at doses well-below the clinically relevant doses that correspond to the LD_50_ for this drug.

**Figure 10:**
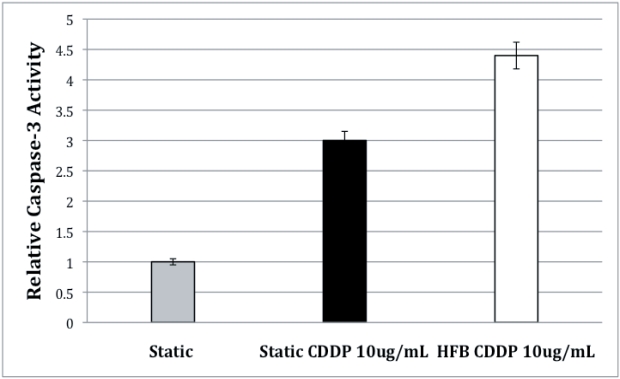
Graphic representation of the relative caspase-3 activity of SAOS-2 cells exposed to CDDP after growth stimulation in simulated hypogravity. SAOS-2 cells grown for 5 days in the hydrofocusing bioreactor and treated with 10 µg/mL of CDDP die by apoptosis as evidenced by a caspases-3 colorimetric assay. The white bar illustrates the caspase-3 activity recovered from SAOS-2 cells grown in the HFB and then treated with CDDP for 24-hours. The black bar illustrates the caspase-3 activity recovered from SAOS-2 cells grown in static normal (1-G) condition and then treated with CDDP for 24-hours, and the grey bar indicates the basal caspases-3 activity of SAOS-2 cells grown in static 1-G condition.

We also investigated the apoptotic effect of the HFB on the SAOS-2 cells by measuring the activity of caspases, using a colorimetric caspases kit that measures the activity of caspases-3 in the samples grown in the HFB for 5 days and treated with 10 µg/mL of CDDP for 24 hours. Caspase-3 activity was measured 24-hours after the chemotherapy treatment. We found that, according to the data previously presented, the SAOS-2 cells cultured in the HFB for 5 days showed an 4.5-fold increase of relative caspases-3 activity detected following CDDP treatment vs. the 3-fold of increased caspases-3 activity detected in SAOS-2 cells treated with the same amount of CDDP (10 µg/mL) in static growth condition. This indicates that the HFB stimulated a CDDP-induced apoptosis of the SAOS-2 cells (**Figure 10**).

The data presented here clearly show that the hypogravity environment greatly sensitized the CD133(+) cells to various chemotherapeutic agents. Importantly, we showed that the CD133(+) cells, which are normally resistant to chemo treatment, become sensitive even at low doses of the noxious drugs.

## Discussion

Modeled hypogravity is a condition in which cells are able to grow in an anchorage independent manner. The hydrofocusing bioreactor HFB from Celdyne, Houston TX was developed by NASA at the Johnson Space Center. The HFB is composed of a 50 mL fluid filled dome, which rotates at a specified speed to provide a unique hydrofocusing capability, that in absence of gas bubbles allows for a low-shear culture environment in which the cells can grow in modeled hypogravity [25]. Another bioreactor that allows cells to grow in a three-dimensional manner is the rotatory cell culture system (RCCS) manufactured by Synthecon (Houston, TX). The RCCS is a 50 mL horizontally rotating culture vessel, which reduces the shear and turbulence generated by conventional stirred bioreactors minimizing mechanical cell damage and simulating aspects of hypogravity [25], [26].

Prolonged exposure of humans and experimental animals to the altered gravitational conditions of space flight has diverse effects on various cellular systems [34], [35], [36], [37], [38], [39]. However, the effects of hypogravity on tumor growth and carcinogenesis are yet unknown. The purpose of this study was to investigate the effects of modeled hypogravity on cancer cell growth.

The existence of cancer stem cells in several solid and hematopoietic tumors has recently been proven [40], [41], [42], [43], [44], [45], [46], [47]. Moreover, it was demonstrated that cancer stem cells are also present in established cancer cell lines [32], [48], [49].

Importantly, it is widely accepted that cancer stem cells are responsible for tumor recurrence after chemo- or irradiation therapy. Although it is still not clear whether the cancer stem cells are derived from original tissue-derived stem cells, bone marrow stem cells, or mature cells that have undergone a dedifferentiation process, it has been suggested that novel strategies for successful cancer therapy should focus on the elimination of cancer stem cells [50].

Because osteosarcoma is a highly resistant tumor to conventional therapies, we investigated whether osteosarcoma cell lines contain a subpopulation of putative stem cells that could be targeted for anti-cancer therapy. One of the biggest obstacles of stem cell research results from the small number of stem cells isolated from tissues. This limits research conducted on normal adult stem cells as well as on cancer stem cells from liquid or solid tumors. Of course, this issue may be overcome by *in vitro* propagation of stem cells (cancerous or not) to obtain a sufficient number of cells to develop novel therapies.

Stem cells have the potential to self-renew and generate a developmental hierarchy of differentiating progeny. The original culture methodology employed by Reynolds et al. [51] and Reynolds and Weiss [52] to show that the adult mammalian brain contains cells that give rise to neurosphere clones has been used to isolate and characterize cells suspected of possessing attributes of stem and progenitor cells. The Reynolds' group showed that the stressful growth conditions of their system due to serum starvation allows dedifferentiation of certain cells, or selects for the most primitive cells by eliminating the differentiated cells that are unable to survive.

In our study, the HFB on day 5 and day 7 increased CD133(+) SAOS-2 cell growth compared to the RCCS vessel and to the earth gravity control. We observed a (+)15-fold proliferation of the SAOS-2 CD133(+) cellular fraction with cells that were cultured for 7-days at optimized conditions in the HFB. The RCCS vessel instead showed a (−)4.8-fold decrease in the CD133(+)cellular fraction respect to the HFB after 7-days of culture. Additionally, 100% of the cells harvested from the HFB were found to be CD133(+), indicating that the HFB had selected for the SAOS-2 undifferentiated cellular fraction. We speculate that the selective growth of CD133(+) cells observed using the HFB vessel could be due to its specific design. The presence of the conic spinner on the axis of rotation of the HFB vessel that hydrofocuses the cell culture allowing for a low-shear environment could be responsible of the selective growth in the HFB environment.

In order to characterize and investigate the cells grown in the HFB, we assessed the expression levels of various adhesion molecules and stem cell markers before and after culturing the cells in simulated hypogravity. The CD133(+) cells appeared healthy and interestingly, both the expression levels and the number of cells expressing several of the stem cell markers other than CD133 were increased after a seven-day run in the HFB. We found that after a seven-day run compared to static growth control conditions there were an increased number of SAOS-2 cells expressing CD133, CD34, CD38, CD117/c-Kit, Sparc, Sox-9, RunX-2, Stro-1, Osteocalcin, Endoglin, Integrin-ß1, and OCT3/4. The fact that we found increased cell number of cells expressing the tested markers and increased overall expression levels of the same after culture of the cells in the hypogravity environment points toward various scenarios. One scenario is that hypogravity could epigenetically regulate the expression of genes involved in cell-cell adhesion and migration, which are expressed during embryogenesis. Other possible scenarios are that hypogravity could affect transcription or post-translational events such as proteasome activity. We are currently analyzing a data set from a gene expression microarray chip in which we studied osteosarcoma cells challenged to grow in HFB versus cells grown in static 1-G in order to better characterize the influence of hypogravity on cellular homeostasis.

The importance of our work is summarized by the unique ability of the HFB to proliferate cancer stem cells and to sensitize them to low doses of chemotherapy. The selection of CD133(+) SAOS-2 sub-population due to cell death of the CD133(−) population is demonstrated by the activation of Caspase-3 activity shown in figure 2 following culture of the SAOS-2 cells (total population) in the HFB for 5-days.

We have also shown that the CD133(+) osteosarcoma cells, which are chemo-resistant to the various drugs, became sensitive to these same drugs at much lower doses than the LD_50_. In this work we choose to test the effects of cisplatin, methotrexate and doxorubicin on osteosarcoma cells, because those are the commonly used drugs to combat this disease [53]. These drugs have different mechanisms of action that can be recapitulated in direct or indirect DNA damage. [54], [55], [56].

Current therapy for osteosarcoma includes neoadjuvant chemotherapy, surgery, and postoperative (adjuvant) chemotherapy [57], [58]. Doxorubicin has been proven effective as adjuvant chemotherapy regimens after surgery in preventing and/or reducing recurrence and metastasis rates in osteosarcoma patients that are operable [58], [59]. Our findings that SAOS-2 cells in general, and CD133(+) SAOS-2 cells in particular, are more sensitive to doxorubicin than to cisplatin or methotrexate helps elucidate some the clinical practice setting. However, more importantly, we have demonstrated the existence of a chemo sensitization switch operated by the hypogravity environment and generated by the unique capabilities of the HFB vessel.

Using the HFB we have formulated a novel technique to select and propagate CD133(+) cells. This will likely prove to be an invaluable tool in furthering stem cell research, embryonic and adult, as well as developing a rapid screening method to test chemotherapeutic agents that are more affective against cancer stem cells. Importantly, we showed that the HFB bioreactor efficiently proliferated CD133(+) cells, while the RCCS rotating vessel did not. Additionally, the data presented in this study clearly show that the hypogravity environment, by stimulating CD133(+) to proliferate, sensitized the CD133(+) to the various chemotherapeutic agents tested.

Presently used chemotherapy drugs have a high rate of failure. Cell culture chemotherapy testing is being used to identify which drugs are more likely to be effective against a particular tumor type in patients. Cell culture drug testing is of value in any situation in which there is a choice between two or more treatments. This includes virtually all situations in cancer chemotherapy, whether the goal is cure or palliation. Often, results are obtained before the patient begins treatment. This kind of testing can assist in individualizing cancer therapy by providing information about the likely response of an individual patient's tumor to proposed therapy. Presently, chemotherapy testing is performed on cancer cells from patients without prior separation and proliferation of the cancer stem cells from the bulk of tumor cells. Knowing which chemotherapy agents the patient's cancer cells or cancer stem cells are resistant to is very important. Then, these options can be eliminated, thereby avoiding the toxicity of ineffective agents. Choosing the most effective agent can help patients to avoid the physical, emotional, and financial costs of failed therapy and experience an increased quality of life.

Importantly, we showed that the HFB bioreactor proliferated CD133(+) cells, which are the minority of the bulk of the tumor cells. It is known that aggressive treatments destroy the majority of the cancerous cells, however a small fraction of the cells, the cancer stem cells, survive and often regenerate tumor cells that are now chemo-resistant [23], [24], [60]. Therefore, to be able to test the efficacy of cytotoxic agents in vitro prior to their use in clinical setting on cancer cells as well as on cancer stem cells may pave the way to more effective chemotherapeutic strategies in patients.

## Materials and Methods

### Cell culture

SAOS-2, HOS and U2OS (human osteosarcoma), T98G and U87MG (human glioblastoma), Du145 and LNCap (human prostate adenocarcinoma), WI38 and H23 (human lung fibroblast and lung adenocarcinoma, respectively), Hep3b (human hepatocarcinoma) and Hela (human cervical cancer) cells were obtained by the American Type Culture Collection (ATCC, Manassas, VA). Mewo and HO-1 cells (human melanoma) were kindly provided by Dr. Paul B. Fisher (Virginia Commonwealth University, VA) and HN12 and HN30 cells (human head & neck squamous carcinoma) were kindly provided by Dr. George Yoo (Wayne State University, MI). Cells were grown in the ATCC-recommended medium (either RPMI 1640 or D-MEM) at 37°C in a water-saturated atmosphere of 95% air and 5% CO2. All media were also supplemented with 2 mM L-glutamine and 10% fetal bovine serum (Hyclone, Logan, UT), 100-µg/mL penicillin, 100-µg /mL streptomycin (Invitrogen, Carlsbad, CA).

### Three-dimensional bioreactor cell culture

A hydrodynamic focusing bioreactor (HFB) (Celdyne, Houston TX) and a rotatory cell culture system (RCCS) (Synthecon, Houston, TX) were used. Both vessels have a volume of 50 mL and a membrane that allows for gas exchange. Culture media, oxygenation, speed, temperature and CO_2_ were kept consistently constant for the two culture systems, which were placed in the same incubator. The vessels can rotate at adjustable speed on a fixed axis. Cells were counted and a range of 1×10^5^–1.2×10^7^ cells were placed in the 40 mL rotating chamber of the HFB for 3- to 7-days set at 25 rpm with airflow set at 20%. For the RCCS, cells were counted and the same number of cells was placed in the 50 mL rotating chamber for 3- to 7-days set at 25 rpm. Cells were then removed and counted again using trypan blue exclusion to determine cellular viability and cell number. The cells were either pelleted for future research or labeled with florescent markers for characterization.

### Cell Sorting

Up to 2×10^7^ cells were sorted either by a magnetic-activated cell sorting (MACS) system, which consists of magnetic beads conjugated to an antibody against CD133 (Miltenyi, Auburn, CA), or by flow cytometry (FACS Aria, BD Bioscience, San Jose, CA). In brief, cells were harvested using 0.25% trypsin, pelleted and labeled with CD133/1 biotin and CD133/2-PE. Cells were washed and labeled with anti-biotin magnetic beads, and then passed through a magnetic column where CD133(+) cells were retained, while unlabelled cells passed through the column. The CD133(+) retained cells were eluted from the columns after removal from the magnet. Positive and negative cells were then analyzed by FACS for purity.

### Flow Cytometry studies

Cells were analyzed by the antigenic criteria using anti-CD133 (prominin1) (Milteny Biotech, Auburn, CA), -CD34 (Milteny Biotech, Auburn, CA); -Sparc, -Sox-9, -RunX-2, -Osteocalcin, -Integrin-ß1, -Endoglin, -Stro-1 (all from SantaCruz, Santacruz, CA), -CD117/c-Kit (BD Bioscience, San Jose, CA), and –Oct3/4 (Cell Signaling, Danvers, MA). Briefly, cells were detached using 0.02% EDTA in PBS and pelleted (10 min at 1,000 rpm), washed in 0.1% BSA in PBS at 4°C and incubated in a solution of 1µg antibody +9 µL 0.1% BSA in 1× PBS. Cells were washed in the same solution once and were processed for sorting (FACS Aria, BD Biosciences, San Jose, CA). Propidium iodide stained cells were also analyzed with a fluorescence-activated cell sorter (FACS Aria, BD Bioscience, San Jose, CA), and the data were analyzed using the flow-Jo cell cycle analysis program (BD Biosciences, San Jose, CA). Cell cycle was analyzed by flow cytometry as follows: following staining with a primary antibody against CD133 (Milteny Biotec, Auburn, CA), and with the secondary antibodies goat anti-mouse (FITC) and mouse anti-goat (PE conjugated) (Santa Cruz, CA), cells were fixed in 4% paraformaldehyde for 30 min at room temperature, washed in PBS, then left for 60 min in PBS/milk 6%. Then cells were stained with a DNA staining solution (0.1% Triton X-100, 0.1% sodium citrate, 1 mg/mL RNAse A and 50 mg/mL propidium iodide) for 2 h at room temperature in the dark. Isotypes and non-probed cells were used as controls. Multiple cell cycle analysis studies were performed to obtain a reproducible model experiments on growing cells.

### Annexin-V assay

Annexin-V was analyzed with the Annexin-V/FITC Kit (Bender MedSystems, Burlingame, CA) following manufacturer's instructions. Cytometry was operated with a FACS Aria (BD Bioscience, San Jose, CA).

### Soft agar assay and sphere assay

Cells were counted and 5×10^3^ SAOS-2 and CD133 (+) and (−) SAOS-2 cells were plated in 6-well culture dishes in 3 mL of soft agar growth medium; 500 µL of medium per well were added every 2 days.

Formed spheres were counted under the microscope. Cells were also plated in ultra-low attachment dishes (Corning, Lowell, MA catalogue# 3262), and the number of spheres for each well was evaluated after 7 and 14 days of culture. The ultra-low attachment surface has a neutral hydrophilic hydrogel coating, which greatly reduces binding of attachment proteins. This minimizes cells attachment and spreading.

### Immunofluorescence

2×10^4^ cells were seeded on two-well micro-chamber slides (Nunc, Naperville, IL). On the day cells were stained, they were washed with PBS and blocked in a mixture of 0.5% BSA, 2 mM EDTA, and FcR blocking reagent (Miltenyi, Auburn, CA) for 20 minutes. The primary antibodies were incubated for 20 minutes on ice, washed with PBS twice, and a secondary anti-mouse or anti-rabbit conjugated with Fitc or PE (e-Bioscience, San Diego, CA) antibodies were used at a dilution of 1∶200 and were incubated on ice for 15 minutes. Negative controls were performed with secondary antibodies only. The slides were viewed under an inverted Olympus IX70 microscope (Olympus America, Inc., Melville, NY). Fluorescence images were captured with Sensicam QE camera (Cooke Co., Auburn Hills, MI) and operated with SlideBook 3.0 software (Intelligent Imaging Innovations Inc., Denver, CO).

### *In vitro* growth characteristics and chemosensitivity

To determine the *in vitro* growth rate of each cell line, 30,000 cells/cm^2^ were seeded in the appropriate tissue culture media. Cells were then harvested and counted by Trypan blue dye exclusion every 24 h, and doubling time was calculated during the exponential phase of growth (from 48 to 96 h after seeding). For saturation density, from day 4 onwards, the medium was changed daily, and cells were counted every 2 days until they stopped growing. LD_50_ was calculated by assessing cell viability using trypan blue exclusion staining and confirmed by MTT assay. Cisplatin (Alexis Biochemicals, San Diego, CA), Doxorubicin (Sigma Aldrich, St. Louis, MO) and Methotrexate (MP Biomedicals, Solon, OH) were dissolved to a stock concentration of 100 mg/mL, 50 mg/mL, and 20 mg/mL, respectively and diluted in normal saline immediately before the experiments. Cells were treated with the chemotherapeutic agent for 1 hour, and then were washed and fresh culture medium was added. Cell viability was assessed 24 hours after treatment. Cells were also treated continuously for 24 hours with the chemotherapeutic agent, and their viability was assessed after treatment. The degree of cisplatin, doxorubicin, and methotrexate resistance was expressed by the drug concentration resulting in 50% inhibition of viability (LD_50_) of the various cell lines. To determine the LD_50_ values 30,000 cells/cm^2^ were seeded in their growth medium containing 10% FBS, and after 24 h the medium was replaced with medium containing 10% FBS and no drug (control) or with different concentrations of the chemotherapy drugs. After 96 h, cells were harvested and counted by Trypan blue dye exclusion to estimate the percentages of cell death compared with the appropriate control, which were then used to calculate the LD_50_ values.

### MTT Assay

1×10^4^ exponentially growing cells were seeded in 100 µL of medium in 96-microwell, flat-bottomed plates. For each of the variants tested, 7 repeats were used. Following 24 hr in culture (to allow the cells to attach and resume growth), 20 µL of different concentrations of the drug were added to each well containing untreated cells. Normal saline was added to the controls. Cells were exposed to the drugs either for 24-hours continuously or for one hour time intervals, as indicated. At the end of drug exposure, the CDDP- MTX or DOX-treated cells as well as parallel control cells were incubated for 4 hours with 50 µL of MTT solution (5 mg/mL). After incubation, culture medium in each well was discarded and replaced with 50 µL of DMSO. After 10 minutes shaking, the absorbance of each well was determined by a spectrophotometer at 510 nm wavelength. The percentage of cell viability was calculated by multiplying the ratio absorbance of the sample versus the control by 100. Chemotherapeutic drugs' LD_50_ was determined as a chemotherapeutic drugs' concentration showing 50% of cell survival as compared with the control cells. The experiments were repeated in seven replicates.

### Caspase-3 colorimetric assay

Caspase-3 activity was measured with a Caspases-3 colorimetric assay kit (Chemicon, (now Millipore, Billerica, MA) following manufacturer's instructions.

### Statistics

The results for each variant in the different experimental designs represent an average of 4 to 5 different experiments. The data of 7 or more measurements were averaged; the coefficient of variation among these values never exceeded 10%. Mean values and standard errors were calculated for each point from the pooled normalized to control data. Statistical analysis of the significance of the results was performed with a 1-way ANOVA.

## References

[1] Reya T, Morrison SJ, Clarke MF, Weissman IL (2001). Stem cells, cancer, and cancer stem cells.. Nature.

[2] Dean M (2006). Cancer stem cells: redefining the paradigm of cancer treatment strategies.. Mol Interv.

[3] Shipitsin M, Polyak K (2008). The cancer stem cell hypothesis: in search of definitions, markers, and relevance.. Lab Invest.

[4] Gao JX (2008). Cancer stem cells: the lessons from pre-cancerous stem cells.. J Cell Mol Med.

[5] Kelly K, Yin JJ (2008). Prostate cancer and metastasis initiating stem cells.. Cell Res.

[6] Mizrak D, Brittan M, Alison MR (2008). CD133: molecule of the moment.. J Pathol.

[7] Tysnes BB, Bjerkvig R (2007). Cancer initiation and progression: involvement of stem cells and the microenvironment.. Biochim Biophys Acta.

[8] Fan X, Salford LG, Widegren B (2007). Glioma stem cells: evidence and limitation.. Semin Cancer Biol.

[9] Farnie G, Clarke RB (2007). Mammary stem cells and breast cancer–role of Notch signalling.. Stem Cell Rev.

[10] Lee CJ, Dosch J, Simeone DM (2008). Pancreatic cancer stem cells.. J Clin Oncol.

[11] Maitland NJ, Collins AT (2008). Prostate cancer stem cells: a new target for therapy.. J Clin Oncol.

[12] Molyneux G, Regan J, Smalley MJ (2007). Mammary stem cells and breast cancer.. Cell Mol Life Sci.

[13] Panagiotakos G, Tabar V (2007). Brain tumor stem cells.. Curr Neurol Neurosci Rep.

[14] Peacock CD, Watkins DN (2008). Cancer stem cells and the ontogeny of lung cancer.. J Clin Oncol.

[15] Zabierowski SE, Herlyn M (2008). Melanoma stem cells: the dark seed of melanoma.. J Clin Oncol.

[16] Wang JC, Dick JE (2005). Cancer stem cells: lessons from leukemia.. Trends Cell Biol.

[17] Soltysova A, Altanerova V, Altaner C (2005). Cancer stem cells.. Neoplasma.

[18] Guo W, Lasky JL, Wu H (2006). Cancer stem cells.. Pediatr Res.

[19] Okamoto OK, Perez JF (2008). Targeting cancer stem cells with monoclonal antibodies: a new perspective in cancer therapy and diagnosis.. Expert Rev Mol Diagn.

[20] Rajan P, Srinivasan R (2008). Targeting Cancer Stem Cells in Cancer Prevention and Therapy.. Stem Cell Rev.

[21] Ren C, Kumar S, Chanda D, Kallman L, Chen J (2008). Cancer gene therapy using mesenchymal stem cells expressing interferon-beta in a mouse prostate cancer lung metastasis model.. Gene Ther.

[22] Lin TL, Fu C, Sakamoto KM (2007). Cancer stem cells: the root of the problem.. Pediatr Res.

[23] Kasper S (2008). Stem cells: The root of prostate cancer?. J Cell Physiol.

[24] Farnie G, Clarke RB (2006). Breast stem cells and cancer.. Ernst Schering Found Symp Proc.

[25] Mihailova M, Trenev V, Genova P, Konstantinov S (2006). Process simulation in a mechatronic bioreactor device with speed-regulated motors for growing of three-dimensional cell cultures.. Ann N Y Acad Sci.

[26] Mitteregger R, Vogt G, Rossmanith E, Falkenhagen D (1999). Rotary cell culture system (RCCS): a new method for cultivating hepatocytes on microcarriers.. Int J Artif Organs.

[27] Sakai S, Mishima H, Ishii T, Akaogi H, Yoshioka T (2009). Rotating three-dimensional dynamic culture of adult human bone marrow-derived cells for tissue engineering of hyaline cartilage.. J Orthop Res.

[28] Villanueva I, Klement BJ, von Deutsch D, Bryant SJ (2009). Cross-linking density alters early metabolic activities in chondrocytes encapsulated in poly(ethylene glycol) hydrogels and cultured in the rotating wall vessel.. Biotechnol Bioeng.

[29] Kumari R, Singh KP, Dumond JW (2009). Simulated microgravity decreases DNA repair capacity and induces DNA damage in human lymphocytes.. J Cell Biochem.

[30] Grun B, Benjamin E, Sinclair J, Timms JF, Jacobs IJ (2009). Three-dimensional in vitro cell biology models of ovarian and endometrial cancer.. Cell Prolif.

[31] Lawrenson K, Benjamin E, Turmaine M, Jacobs I, Gayther S (2009). In vitro three-dimensional modelling of human ovarian surface epithelial cells.. Cell Prolif.

[32] Gibbs CP, Kukekov VG, Reith JD, Tchigrinova O, Suslov ON (2005). Stem-like cells in bone sarcomas: implications for tumorigenesis.. Neoplasia.

[33] Tirino V, Desiderio V, d'Aquino R, De Francesco F, Pirozzi G (2008). Detection and characterization of CD133+ cancer stem cells in human solid tumours.. PLoS One.

[34] Badhwar GD, Nachtwey DS, Yang T-H (1992). Radiation issues for piloted Mars mission.. Adv Space Res.

[35] Baisden DL, Beven GE, Campbell MR, Charles JB, Dervay JP (2008). Human health and performance for long-duration spaceflight.. Aviat Space Environ Med.

[36] Barr YR, Bacal K, Jones JA, Hamilton DR (2007). Breast cancer and spaceflight: risk and management.. Aviat Space Environ Med.

[37] Dicello JF (2003). The impact of the new biology on radiation risks in space.. Health Phys.

[38] Durante M, Cucinotta FA (2008). Heavy ion carcinogenesis and human space exploration.. Nat Rev Cancer.

[39] Williams JR, Zhang Y, Zhou H, Osman M, Cha D (1999). Predicting cancer rates in astronauts from animal carcinogenesis studies and cellular markers.. Mutat Res.

[40] Al-Hajj M, Wicha MS, Benito-Hernandez A, Morrison SJ, Clarke MF (2003). Prospective identification of tumorigenic breast cancer cells.. Proc Natl Acad Sci U S A.

[41] Chiba T, Kita K, Zheng YW, Yokosuka O, Saisho H (2006). Side population purified from hepatocellular carcinoma cells harbors cancer stem cell-like properties.. Hepatology.

[42] Collins AT, Berry PA, Hyde C, Stower MJ, Maitland NJ (2005). Prospective identification of tumorigenic prostate cancer stem cells.. Cancer Res.

[43] Hamburger AW, Salmon SE (1977). Primary bioassay of human tumor stem cells.. Science.

[44] Singh SK, Clarke ID, Terasaki M, Bonn VE, Hawkins C (2003). Identification of a cancer stem cell in human brain tumors.. Cancer Res.

[45] Wodinsky I, Swiniarski J, Kensler CJ (1968). Spleen colony studies of leukemia L1210. 3. Differential sensitivities of normal hematopoietic and resistant L1210 colony-forming cells to 6-mercaptopurine (NSC-755).. Cancer Chemother Rep.

[46] Dome B, Timar J, Dobos J, Meszaros L, Raso E (2006). Identification and clinical significance of circulating endothelial progenitor cells in human non-small cell lung cancer.. Cancer Res.

[47] Grichnik JM, Burch JA, Schulteis RD, Shan S, Liu J (2006). Melanoma, a tumor based on a mutant stem cell?. J Invest Dermatol.

[48] Hirschmann-Jax C, Foster AE, Wulf GG, Nuchtern JG, Jax TW (2004). A distinct “side population” of cells with high drug efflux capacity in human tumor cells.. Proc Natl Acad Sci U S A.

[49] Kondo T, Setoguchi T, Taga T (2004). Persistence of a small subpopulation of cancer stem-like cells in the C6 glioma cell line.. Proc Natl Acad Sci U S A.

[50] Dean M, Fojo T, Bates S (2005). Tumour stem cells and drug resistance.. Nat Rev Cancer.

[51] Reynolds BA, Tetzlaff W, Weiss S (1992). A multipotent EGF-responsive striatal embryonic progenitor cell produces neurons and astrocytes.. J Neurosci.

[52] Reynolds BA, Weiss S (1992). Generation of neurons and astrocytes from isolated cells of the adult mammalian central nervous system.. Science.

[53] Schuetze SM (2007). Chemotherapy in the management of osteosarcoma and Ewing's sarcoma.. J Natl Compr Canc Netw.

[54] Reedijk J, Lohman PH (1985). Cisplatin: synthesis, antitumour activity and mechanism of action.. Pharm Weekbl Sci.

[55] White JC (1981). Recent concepts on the mechanism of action of methotrexate.. Cancer Treat Rep.

[56] Sartiano GP, Lynch WE, Bullington WD (1979). Mechanism of action of the anthracycline anti-tumor antibiotics, doxorubicin, daunomycin and rubidazone: preferential inhibition of DNA polymerase alpha.. J Antibiot (Tokyo).

[57] Berend KR, Pietrobon R, Moore JO, Dibernardo L, Harrelson JM (2001). Adjuvant chemotherapy for osteosarcoma may not increase survival after neoadjuvant chemotherapy and surgical resection.. J Surg Oncol.

[58] Ferguson WS, Goorin AM (2001). Current treatment of osteosarcoma.. Cancer Invest.

[59] Zalupski MM, Rankin C, Ryan JR, Lucas DR, Muler J (2004). Adjuvant therapy of osteosarcoma–A Phase II trial: Southwest Oncology Group study 9139.. Cancer.

[60] Al-Romaih K, Somers GR, Bayani J, Hughes S, Prasad M (2007). Modulation by decitabine of gene expression and growth of osteosarcoma U2OS cells in vitro and in xenografts: Identification of apoptotic genes as targets for demethylation.. Cancer Cell Int.

